# Future public health emergencies and disasters: sustainability and insights into support programs for healthcare providers

**DOI:** 10.1186/s12888-022-04309-z

**Published:** 2022-10-27

**Authors:** Misha Dhuper, Lesley Ruttan, Lindsey MacGillvray, Martha McKay, Adrienne Li, Donna Stewart, Susan Abbey, Suze Berkhout, Kathleen Sheehan, Christian Schulz-Quach

**Affiliations:** 1 Department of Psychiatry, Temerty Faculty of Medicine, 1 King’s College Cir, Toronto, ON M5S 1A8 Canada; 2 Toronto Rehab, University Health Network, 550 University Avenue, Toronto, ON M5G 2A2 Canada; 3 Centre for Mental Health, University Health Network, 190 Elizabeth St, Toronto, ON M5G 2C4 Canada; 4 Department of Psychiatry, University of Toronto, 250 College Street, Toronto, ON M5T 1R8 Canada; 5 Princess Margaret Cancer Centre, University Health Network, 620 University Ave, Toronto, ON M5G 2C4 Canada

**Keywords:** Work and Mental Health, COVID-19 pandemic, Mental health supports, Healthcare workers, Workplace-based programs, Healthcare delivery, Mental health provider, Disaster, Emergency

## Abstract

**Background:**

The mental health of healthcare workers (HCWs) has been at the forefront throughout the COVID-19 pandemic. While workplace-based support programs have been developed in hospitals globally, few systematically collected data. While critical to their success, information on these programs and the experience of mental healthcare providers (MHP) who support colleagues is limited. The objective of this study was to explore the experiences of MHP caring for HCW colleagues within a novel workplace-based mental health support program during the COVID-19 pandemic, to provide insights on facilitators, areas for improvement and barriers to program sustainability.

**Methods:**

This qualitative study used semi-structured interviews conducted by videoconference between September 2020 to October 2021. UHN CARES (University Health Network Coping and Resilience for Employees and Staff) Program was developed during the first wave of the COVID-19 pandemic in March 2020. It supports over 21,000 staff members within the UHN, Canada’s largest academic health research institution, in Toronto, Canada. Purposive sampling was used to select 10 of the 22 MHP in the UHN CARES Program (n = 10). Using a critical realism framework, key components required to sustain a successful workplace-based mental health support program for HCWs and balance the needs of MHP were determined.

**Results:**

Six psychiatrists and four psychologists (n = 10) with varying roles at UHN participated in 17 interviews, including seven repeat interviews exploring changes over time within the pandemic and program. Components which facilitated the success of the program included flexibility in scheduling, confidential health record storage, comprehensive administrative support, availability of resources and adaptive quality improvement approach. Recommendations for improvement included opportunities for peer supervision, triaging of cases, and managing HCW expectations. MHP found caring for HCWs to be meaningful and they utilized existing clinical skills during sessions. Challenges included working in a virtual setting, navigating boundaries when caring for colleagues, and managing the range of service users and their needs.

**Conclusions:**

These findings suggest how support programs can be structured for HCWs, how to provide support, and how to sustain this support, allowing health systems to balance the needs of HCWs and MHPs in preparation for future public health emergencies.

**Supplementary Information:**

The online version contains supplementary material available at 10.1186/s12888-022-04309-z.

## Background

During previous coronavirus outbreaks, such as the 2003 SARS-CoV-1 outbreak in Toronto, Canada and 2014 MERS-CoV outbreak in Jeddah, Saudi Arabia, healthcare workers (HCWs) experienced emotional distress centered around inadequate institutional emergency preparedness [1, 2], as well as personal safety and the safety of their loved ones [3]. These serious and long-lasting mental health effects of pandemics on HCWs have long been recognized [3–6]. As such, hospitals rapidly developed and implemented workplace-based programs to support the mental health of their HCWs with the emergence of the COVID-19 pandemic in the spring of 2020 [7]. However, there was little evidence to guide how best to provide this care [8–10]. These new programs were often staffed by mental healthcare providers (MHPs) at the same institution, requiring them to provide support to colleagues [8]. While several studies have described these programs, there is limited data on the experience of the MHPs in these programs [11].

The COVID-19 pandemic has now been ongoing for two years. Early hopes that the pandemic would be short-lived are gone and there is increasing recognition that it will continue, even at endemic levels [12]. New waves of infectious variants have spread globally, causing cases and distress among HCWs to peak repeatedly [13]. While the concerns underlying this distress have shifted throughout the pandemic, from worries about availability of appropriate personal protective equipment (PPE) in the early waves to stress about cancellations of surgeries and staffing shortages in later waves, there is increased recognition that HCWs will need ongoing support to cope now and in the future [14].

These shifting stressors impact HCWs and the MHPs staffing workplace-based programs. Understanding the experiences of MHPs is critical if these programs are to continue to support HCWs [15]. While one study reported the experience of MHPs supporting HCWs during COVID-19, this was not focused on those within a workplace-based program and was not embedded within the frame of a programmatic analysis [11]. In this paper, we used qualitative analysis to explore the experiences of psychologists and psychiatrists who provided mental healthcare to HCWs during the COVID-19 pandemic through the UHN CARES (University Health Network Coping and Resilience for Employees and Staff) Program in Toronto, Ontario. Embedded within a longitudinal program evaluation, we aimed to investigate the challenges and benefits of caring for HCW colleagues in a workplace-based mental health support program; understand strengths and weaknesses of the program; and determine the aspects of the program which contributed to its success and sustainability.

## Methods

We conducted semi-structured interviews with MHPs who provided individual psychological or psychiatric care to HCW colleagues. The study followed COREQ reporting guidelines [16]. It was approved by the UHN Quality Improvement Review Committee (QIRC) (QI-ID: 20–0069) and exempt from Research Ethics Board (REB) review. Informed consent was obtained from all participants.

### Setting

UHN is Canada’s largest academic health research institution with two general hospitals, a comprehensive cancer centre, a health education institute, and a multi-site rehabilitation institute. It has over 21,000 staff members, many of whom provided care during the SARS outbreak in 2003 [19]. Since the start of the pandemic, over 2400 patients with COVID-19 have been admitted to UHN, primarily to intensive care and dedicated medical units. UHN is a provincial resource for extra-corporeal membrane oxygenation (ECMO) and provides some of the highest acuity medical care to COVID patients; the institution had also been tasked with supporting a number of under-resourced long-term care (LTC) facilities within the greater Toronto area (GTA), which contributed to the need for large-scale work-related redeployment amongst UHN employees from clinical and non-clinical areas.

**Fig. 1:**
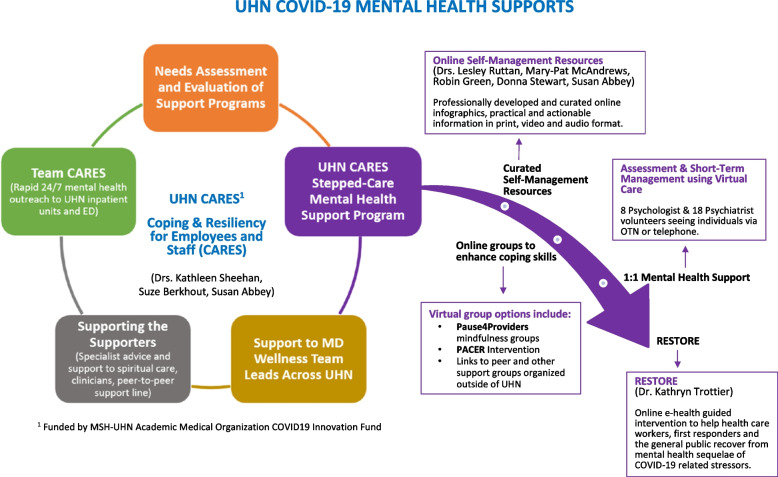
Overview of UHN COVID-19 Mental Health Supports

As shown in Fig. 1, the UHN CARES Program was rapidly developed and implemented in April 2020 as a modified stepped care model, with a hierarchy of interventions matched to the individual's needs [17]. It complemented existing institutional wellness initiatives, such as the Employee Assistance Plan (EAP), a peer-to-peer support phone line, and drop-in respite centres [18]. The two highest level intervention steps enabled HCWs to self-refer for individual mental health care, which was provided by UHN psychologists and psychiatrists who offered to take on HCW colleagues as clients during the pandemic. A novel and critical aspect of the design was the embedding of a quality improvement approach, which used a logic model and quantitative and qualitative methods for program evaluation.

### Participants

Purposive sampling was used, with researchers inviting specific providers for interviews to ensure a broad range of MHP experiences. The criteria included those who saw many versus fewer referrals, saw these referrals at different points in the pandemic, employed a varied number of sessions with HCWs and had a range of years in practice (Table 1). Seven participants were selected between September 2020 and March 2021 and three more were added between July 2021 and October 2021 to ensure an adequate variation of the aforementioned criteria. As a result, of the 22 total MHPs participating in UHN CARES, 10 were selected and agreed to participate in the one-on-one interviews.

**Table 1:** Participant Characteristics

Role	Years in Practice	# of patients seen	# sessions per HCW service users (range)	Timeframe for Provision of Clinical Care
Psychiatrist	2	7	1 to 13	First wave and early second wave
Psychiatrist	7	4	3 to 11	First and third wave
Psychiatrist	15	10	1 to 11	All waves
Psychiatrist	6.5	5	1 to 7	First and third wave
Psychiatrist	31	2	1 to 27	First wave
Psychiatrist	4.5	2	2 to 3	First and third wave
Psychologist	2.5	9	4 to 23	All waves
Psychologist	21	64	1 to 39	All waves
Psychologist	32	23	1 to 9	All waves
Psychologist	8	2	7 to 9	All waves

### Data collection

**Fig. 2:**
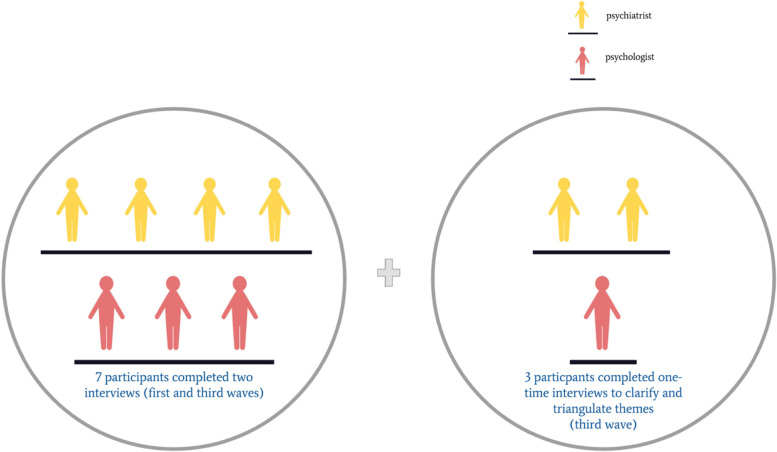
Number of participants recruited in the two phases of the study

A semi-structured interview guide was developed to explore MHPs’ experiences with UHN CARES, their perspectives on the mental health needs of HCWs in the pandemic, how to best meet those needs, and opportunities for program improvement. Interviews were conducted by multiple study authors (KS, MD, SB) by videoconference, each 40–50 min. Each interviewer maintained a record of reflexive notes relating to their interviews and interviews were discussed amongst the researchers to capture immediate impressions after each took place. Interviews were audio recorded and transcribed verbatim. Seven interviews were conducted between September 2020 and March 2021 and an interim thematic analysis was conducted. The same MHP participants were re-interviewed between July 2021 and October 2021, with an additional three MHP interviews during this time to clarify and triangulate themes such as the evolution of the CARES users throughout the various waves of the pandemic, the navigation of boundaries between MHPs and HCWs, MHP coping strategies, MHP learning and growth as well as recommendations and feedback for the UHN CARES program (Fig. 2). Interview prompts were modified iteratively, and field notes were made after the completion of the interviews.

### Data analysis

The larger program evaluation, which combined quantitative and qualitative methods, used a framework of critical realism (CR) to have continuity between both aspects of the evaluation. CR identifies strength in quantitative analysis for the exploration of empirical regularities, and in qualitative methods for illuminating novel, complex relationships not captured by the predetermined categories of quantitative measures [19, 20]. Transcripts underwent close reading by multiple team members and a data-driven codebook was created using a reflexive interpretive thematic analysis that took place within a collaborative process of discussion and reflection amongst MD, SB, KS and CSQ. All transcripts were coded into the provisional coding frame (NVivo 12 Plus) by MD, KS and SB, which was refined with subsequent transcripts, and then finalized. To enhance the reliability of the analysis, developing themes were presented to the larger group of mental health providers at two different peer support meetings and feedback relating to themes was incorporated in subsequent iterations of analysis. The project followed the consolidated criteria for reporting qualitative research (COREQ) guidelines [16].

## Results

We conducted 17 interviews with four psychologists and six psychiatrists. Sampling was stopped when data saturation was reached.

Table 1 shows participant characteristics and program engagement. While all participants worked at UHN, their responsibilities varied during the pandemic depending on their re-deployment status. Only one psychologist was fully re-deployed to UHN CARES, with most maintaining a substantive portion of their pre-pandemic role in addition to their work in UHN CARES. We were not able to quantify the number of hours MHPs had available for the program because most did not specifically reserve times in their calendars for UHN CARES referrals. Instead, whenever there was a callout to the group about a new referral, MHP with availability matching the patient’s availability would take the referral.

As shown in Table 2, major themes from the data included: successful program characteristics, opportunities for improvement, positive and challenges aspects of MHP experience, and strategies for clinical engagement.

**Table 2:** Themes Developed from Health Care Provider Qualitative Evaluation

Theme	Subtheme	Example Quote(s)
1. Successful Program Characteristic	Flexibility of scheduling and clinical care	*“I’ve taken on what I could, given caseloads…the flexibility to be able to schedule when I can, and when that individual's needed… and the clinical flexibility to decide, even though it designed, or intended to be fairly brief, but if individuals needed something additional, the flexibility in the decision for myself just to say, okay, I’ll continue to see them until things have improved, or until there's, you know, somewhere else they can access.”* PR-interview-9 (Psychologist) *“Because I felt I was in a situation of flexibility and availability, I saw patients often later in the evening…Which was highly appreciated by patients. So, you know, for the sheer fact that they were typically working shifts, or they were working during the day, to be able to see a psychiatrist out of hours was really, really appreciated… If you're really focusing on mental health and not just wellbeing, I think there needs to be enough flexibility to offer this kind of care when the employees need it and not just when we are available.”* PR-interview-3 (Psychiatrist) *“The good thing about UHN Cares and doing it from home is that I talked to people sometimes after 5:00 or I talked to them on the weekend, and that is very helpful for people who work full-time. So, I think that that was a good thing, that we were able to be flexible about that.”* PR-reinterview-7 (Psychologist)
	Healthcare record keeping and confidentiality	*“People were definitely concerned about confidentiality. And they were very relieved to know that our process in the Cares program was set up so that… their clinical charts was completely separate from any kind of documentation within UHN… I think that was something that almost everyone had said that they felt reassured by…I don't think anyone was worried so much about the one-on-one, interpersonal component of confidentiality. It was more, like, where was their information being housed? Who could have access to it? And that being very separate from, like, the hospital's electronic medical record system, I think, was something that most people found very reassuring.”* PR-reinterview-1 (Psychiatrist) *“There was some question as to who sees it, who has access to the documentation, does it go into EPR, and you know, all of the answers to that were no, it does not, and it's only seen by the UHN Cares team.”* PR-interview-9 (Psychologist)
	High quality administrative support	*“…then being able to just email the [template] PDF so that [the admin] could file them was really useful. And having the admin support for booking, which I haven't historically always had was really helpful. The people running the program are excellent…The billing was good… And then if I wanted to refer, like when the one person I’m working with now I did want her to be able to access more intensive therapy and [the admin] just said, oh, sure, we can, you know, refer to the psychology team, and I was like, oh, that's easy. So, it was just, you know, it was great!”*PR-interview-4 (Psychiatrist) *“The templates were really helpful to use, like everything was sort of set up, I didn't really have to wonder, or worry was my record keeping good enough? Where was I going to keep the charts? Like, all of those kinds of things that you need to make sure that you're doing to be in good medical-legal compliance, it was taken care of, especially because I don't actually typically see outpatients, and I don't have a process for any of that sort of thing. It was very helpful to have it all set up for me. It made me much more comfortable and confident in doing the work and felt very good about doing it. I knew I wasn't going to get either bogged down in something, like paperwork, but I also knew I wasn't going to inadvertently make some kind of mistake, because again, I'm not used to following outpatients. So, I didn't have to worry about that.”* PR-interview-1 (Psychiatrist)
2. Challenges within Program/ Recommendations for Change	Increase opportunities for peer supervision	*“I think I’m in a special position because I have a supervisor. I guess peer supervision would be nice, because sometimes I ask my supervisor what types of cases she has been dealing with and whether there are any similarities. How does she deal with this particular type of issue? So, I feel like maybe some type of group discussion can be quite helpful because there might be recurring themes, or you could get ideas from what other people are doing. And obviously, most of the people are more senior than me so I’m going to be learning from them as well.”* PR-interview-2 (Psychologist)
	Enhanced triaging of CARES Users	*“I don't think [I felt pressured to see people really quickly]…I think the idea at the beginning was that we would see them within a certain period of time. So, that's okay, I mean, if I was available, I could do it. But I think if I knew it was somebody who was less urgent, I would schedule them in my clinical time, I wouldn't have seen them on a weekend, or on my research day. Like, I would have scheduled them like a few weeks out, into a clinical spot.”* PR-interview-4 (Psychiatrist)
	Managing Expectations of CARES Users	*“Where it's been really challenging, and then they also feel, I’ve had to deal with a fair number of calls with people who'd hoped that we would help their family members… particularly adolescents with eating disorders and anxiety… that couldn't access resources in other places, or husbands or wives. So, needing to be able to say, no, no, we can't.”* PR-interview-11 (Psychiatrist) *"I think as the discussion progressed, my sense was that there was a hope that UHN would be able to provide a regular therapist that this individual could see weekly…it went sort of badly in that situation then having to sort of set the boundary and say, well, actually, you know, weekly therapy is outside of the scope of what this program is intended to provide. So, here's what I’ll be able to do, and you know, in the circumstance, whether it was in the context of the Cares program or our regular outpatient psychiatry clinics, as you probably know, securing long-term psychotherapy is not an easy thing to do. So, it was also managing expectations in that regard to say I can see what I can do, to see if my colleagues have any names that they would recommend. It would be up to you, as the individual, to reach out. And essentially, there are no guarantees in this process, it often takes a lot of effort and work to secure that.”* PR-interview-10 (Psychiatrist)
3. Positive Aspects of Provider Experience	Finding meaning, purpose, and motivation in clinical work during the pandemic	*“It's a good feeling that I'm part of this, that I'm helping out in some way, other than my regular work, like this is kind of getting my feet and hands kind of wet, or whatever the saying is, like right in there, right? With them. It's a good feeling, a camaraderie, that we're all working towards something. I guess it's kind of stretching a bit, which was nice, even though it wasn't in my comfort zone to get a little bit of a new skill set of working with health care providers and seeing what that's like. And yeah, I think, maybe because I could identify a little bit, especially with the last one that I got to know a little bit more over time, I think I could identify with her because we were both… it felt more like we were parallel, like on equal ground rather than I was, like, her… even though I was there, kind of, doing psychotherapy, it felt a little bit more equal in this role of the Care program. And I think that I, you know, even though we were only there for a few sessions together, I felt really touched at the end, you know, that I was working with her to get her somewhere professionally and also personally, and that was helping others… I don't know. It just felt like a really touching, important feeling for me.”* PR-interview-6 (Psychiatrist)
	Familiar clinical care issues within scope of expertise	*“I see, you know, a wide range of people in my private practice, you know, and I'm also a neuropsychologist, so I've worked with people, you know, with a really wide range of brain injury for a long, long time. So, you know, as much as it's different, it's also the same, you know, it's people who have lived through something traumatic, life changing, that has really impacted. So, yeah, I would say that, you know, working with these people, I would definitely be drawing on similar kinds of skills that probably I would be, that I've also used with private clients or even patients on the brain injury program too.”* PR-reinterview-5 (Psychologist)
4. Challenges within Provider Experience	Providing care in virtual settings	*“I hadn't been used to the virtual environment, but I’ve got to like it. At first, I found it quite hard, I don't know, because it doesn't feel, it doesn't feel… you don't get the sort of same affective cues necessarily that you get with being somebody. I can't really feel whether they're anxious or depressed, like it's quite hard to tell. And I probably have a touch of ADHD, so I found it a bit harder to, like as I’m talking to you now, I’m trying to sort of, because of the way that the devices are positioned, my eyes are drawn to you, but I should be looking up at the camera…The first two or three months, i just found it exhausting, I would just go home and go to bed.”* PR-interview-11 (Psychiatrist)
	Navigating and maintaining boundaries in shared work setting	*“Yeah, we're colleagues, we're peers, but they're also coming to me in a role where it's kinda, I don't want to say they're beneath me, but it's kind of like I’m there to help them more of an authority on how I can be helpful. I'm not used to that. And yeah, I guess that did come up once or twice, where what if we do, you know, they're changing locations, what if we do, at some point, meet in a professional realm, yeah. That might be a bit messy too, potentially.”* PR-interview-6 (Psychiatrist) *“I think what was complex, again, going back maybe to this we're all in this together experience, was I was taking care of highly, highly, at times, not all of my patients, but some, three examples come to mind, of highly distressed, dysregulated, desperate colleagues, who were very close to my own identity. And were expressing concerns that were close to my own experiences, only the amplitude was different. And so, I think what made it more difficult, if I reflect upon it, was the level of identification and the degree of identification, and projection, that was going on.”* PR-reinterview-3 (Psychiatrist) *“I was happy to be able to help people who work at UHN as well. More just having to be a little more careful around boundaries and just thinking about, you know, not disclosing a lot more of my own experiences than I maybe normally would for seeing patients, just because I do have more in common with the people I was seeing through the Cares program. And especially for ones who maybe worked where I would come across them, just talking about, well, what would you like me to do if I see you at the hospital? So, just being a little bit more mindful of that.”* PR-interview-4 (Psychiatrist) *“I think we did a pretty good job because there were times, for example, where [admin1] or someone, I worked at, you know, if anyone from my location was looking for care, which happened several times, I wouldn't, you know, [admin1] would make that clear when she sent the message out to the psychologists to say, you know, nobody from here. So, I would always know, okay, that person, you know, that would be someone I know. I think because UHN is so big, you know, it really is, like, separate, with the different hospitals and things. So, it really did quite separate and pretty unlikely that I'm going to, you know, be bumping into any of these people in the halls. So, it actually worked fine.”* PR-reinterview-5 (Psychologist) *“There was some concern in close proximity to my clinical role, versus the people I was seeing, but we were able to manage that well. So, knowing that we are in close proximity, potentially could see each other, potentially, if there's any sort of inter-program movement with patients, that we could potentially work with each other in that capacity. So, that was discussed in the first session, and had a plan confirmed and the person was comfortable with continuing. Had they not been, I would have just said, okay, let's find you someone else. But they were comfortable with that. So, we sort of set the ground rules and moved forward with that.”* PR-interview-9 (Psychologist)
	Managing requests, referrals, follow-up care outside of the workplace program	*“It was initial validation, support, normalization of this experience, talking about problem solving versus navigating things with the workplace and with their partner and some hygiene kinds of interventions, self-care kinds of interventions… I think they were somebody who had had a lot of experience of actually doing psychotherapy or psychodynamic types of therapies but had never fully explored this before, and then all of the pandemic-related stressors really brought things to the forefront. And sort of compelled this individual to think about doing that on a regular basis, and so had asked for some recommendations or names, whether or not they reached out, I don't know. But I contacted some of my colleagues to get a name or two to provide to this individual, and they seemed to be very satisfied with that.”* PR-interview-10 (Psychiatrist) *“I think it’s quite challenging because one, it’s remote therapy and then two, often they don’t have much capacity to do a lot of stuff outside of their work. I think often people are exhausted or it’s like their life has been all consumed by work and by COVID. I think CBT, especially, is like a type of therapy that can involve a of homework, a lot of them going away and doing exercises, trying out strategies. And I think, sometimes that’s just not practical for some people, so sometimes the sessions have been more maybe helping them understand. Or helping them have a place to ventilate and express their feelings and to understand things maybe within a CBT framework, or trying some really simple stuff like mindfulness and sleep strategies. So, nothing really very involved in terms of the CBT. And obviously with COVID it’s just hard to do a lot of the stuff that we would usually recommend, which is like, go socialise or do hobbies. There’s a lot of stuff that you would normally suggest in therapy that is not possible because of their job, but as well because of all the lockdowns and restrictions. So, I think we’ve had to adapt a lot and be really, obviously, flexible, and also be really clear about what’s achievable and what could help the most in this situation. But it might not be a goal normally in therapy, it might be setting different goals for this situation and you adapted to the person.”* PR-interview-2 (Psychologist)
	Anticipation of support required	*“Initially, recognizing that this is overall, a relatively healthy, well population. And thinking about, like, I didn't have to worry so much about all the worst-case scenarios that could be going on. So, I think getting used to that was… well, one area of challenge that, like, there might be a crisis happening right now for this person but overall, they have ways of coping. And it's not on me to have to do these big interventions, that we can just help them get through, help them reconnect with their own ways of coping and I think being able to get used to that was… to some degree, I guess, a challenge, that I didn't have to react and respond in the way that I might react or respond to an acute situation with, say, an inpatient, that kind of thing. That it was a different ballgame altogether. And I think that was probably… And that, again, it felt like this sort of thing that it was a benefit to me too, because it sort of stretched me and stretched my skills in different kinds of ways. So, what maybe started as a challenge, I think, ultimately, was very beneficial.”* PR-interview-1 (Psychiatrist) *“It was a bit frustrating but more… frustration comes from when you have expectations that aren't met, and I think it's kind of like I'm not sure what to expect but yeah, it was a bit frustrating because I guess what I wanted was to be helpful and I wasn't sure if I was being helpful, right? So, it's kind of like, here's a bunch of things I'm throwing at you, and you're not really giving me much feedback. So, this doesn't feel so great, I don't know…I could imagine a lot of people don't really know exactly what they're wanting, they're just suffering, right? They're stressed.”* PR-interview-6 (Psychiatrist)
5. Strategies for Providing High-Quality Care	Skills and tools for patient-centredness	*“And I think that for a lot of people, my experience was that even when there was what had felt to them to be more distress and more of a crisis, knowing that they had someone to connect with and that they were going to be connecting with them quickly and in my case, I told people that they could set the pace of how often they wanted to be seen, and how many follow ups they would have. I think that for a lot of people, that sort of helped settle, like they knew that someone was there to coach them. And that's really what a lot of the work, I think, that I was doing was. I would sort of conceptualize it much more in a kind of coaching frame, more than the high acuity psychiatric patients who I typically see on the inpatient unit. This is a lot more coaching and sort of helping people see the kinds of coping skills and see their own resiliency that they had, that they just weren't really as in tune within the present moment.”* PR-interview-1 (Psychiatrist)
	Personal coping strategies	*“We were all going through this. All of us. This is not a situation where I'm treating someone because I studied it, because I'm a resident, I learn how to deal with it, I’m a staff, I keep developing skills and then I'm taking care of a disorder. No, this was a situation where we were all going through the same anxieties and the same uncertainties, and the same sense of overwhelm, exhaustion and sense of loss of safety and orientation. And I remember that it was sometimes hard to be the containing, holding space for distressed people when my own distress wasn't insignificant. And yet, at the same time, this sense of being able to provide help to others was also, of course, a way of stabilizing my own self-esteem and my own sense of integrity.”* PR-reinterview-3 (Psychiatrist) *“I try and have a routine. So, I think it's quite important—like, I haven't been doing it that well, but trying to have good boundaries around working and not working because for me, like, I’m working mostly from home. So, so I think it's easy to not have that division. So, you know, like, checking email at all hours and then I work in research as well, so research literally people are on like 24/7, they're answering emails 24/7. So, I try and like not do that. So, I make sure that I have the downtime and then, like, obviously, try and get enough sleep. I think sleep is really important. Try and get some physical activity in. And then also, like, take a bit of time off. So, recently, we travelled for the first time. So, I think that that really helps, so kind of change of setting, yeah.”* PR-reinterview-2 (Psychologist) *“I think in the beginning it was sort of a sense of guilt that, you know, some questioning of was I doing enough? Was I doing enough for the Team Cares program? Could I be making more of an effort in this sphere or that sphere? So, there was a little bit of room through there. You know, it also was, as with any time we set a boundary that's important for one's own time and sanity, there's that doubt sort of intertwined with relief of having set a boundary. It helped that it felt like I was contributing in other ways…You know, where I left things was if you're ever in a pickle and there aren't enough providers or things like that, please don't hesitate to reach out. But if you've got a good crew and they're able to meet the needs of the patients that are coming, then that freed me up to do some other things that were also important.” PR-interview-10 (Psychiatrist)*

### Successful program characteristics

#### Flexibility of scheduling and clinical care

As the pandemic evolved and MHPs returned to their normal duties, the program offered sufficient flexibility for providers to titrate their caseload individually (Table 1). This control over caseload was viewed positively as some MHPs expressed feelings of guilt because they were not able to accept many referrals. There was also flexibility in the scheduling of appointments, as HCWs were often seen during evenings and weekends. MHPs reported that this was also appreciated by HCWs, contributing to the patient-centeredness of the program.

#### Healthcare record keeping and confidentiality

MHPs were concerned about record keeping and reported HCW concern about confidentiality. The program’s solution of having consultation and progress notes maintained separate from the electronic health record was appreciated, even though this deviated from usual patient care practice.

#### High quality administrative support

Reliable administrative support made MHPs feel more at ease when taking on referrals. A single administrative assistant managed referrals, triaging, record keeping, communication with HCWs and bridged transitions of care between providers. The administrative assistant also managed a MHP webpage with all relevant documents (e.g., templates for letters to primary care providers) and other resources. Online clinical and community resources used by individual providers were shared amongst MHPs with the help of the program’s administrative staff. These were especially helpful for those not routinely involved in outpatient practice.

### Challenges within program/ recommendations for change

#### Increase opportunities for peer supervision

Some MHPs recommended the addition of peer supervision to the program to ensure quality care and enhance cohesion and mutual support amongst the providers. Optimal frequency was identified as approximately quarterly, given the demands on time from other clinical and administrative obligations, which increased as various pandemic-related restrictions were eased. This was implemented following wave 1 and some noted that seeing this modification to the program occur provided confidence in the process of continuous quality improvement.

#### Enhancing triaging of CARES Users

UHN CARES was intended to be an easy-to-access, short-term service with a rapid response time to first appointment. Some MHP suggested that modified triaging would enable them to meet with acute users more quickly, whereas less acute users could potentially be seen at a farther time point, rather than MHPs feeling obligated to schedule into their personal time.

#### Managing expectations of UHN CARES Users

Clarifying and managing the expectations of UHN CARES users was identified as a challenge. The scope of the CARES program was limited to staff working at UHN, with distress relating to COVID-19. Challenges involved users’ presenting issue being primarily focused on long-standing mental health challenges or family members. While providers did their best to connect those users to community resources, they reported that improving communication of scope and limitations of UHN CARES would be helpful.

### Positive aspects of provider experience

#### Finding meaning, purpose, and motivation in clinical work during the pandemic

Even though it increased workload, MHPs found the experience of providing care to HCWs to be meaningful. Some even found the experience therapeutic and purposeful as they felt that they were contributing to pandemic-related mitigation efforts.

#### Familiar clinical care issues within scope of expertise

Although prior to providing care, several wondered about being equipped to help HCWs cope, MHPs reported using skills developed in previous clinical work and were pleased it transferred to the pandemic setting. Some MHPs felt that UHN CARES service felt less complex than their usual day-to-day work and that they felt prepared to manage whatever issues arose. Users accessing the program presented with a wide range of concerns, including the inability to cope with the pandemic and its various stressors (e.g., redeployment, institutional policy changes, political tensions, etc.) and exacerbations of pre-existing mental health illnesses. Pandemic-related restrictions (e.g., access to hobbies, social contact, etc.) meant MHPs had to accommodate and be creative with their treatment recommendations.

### Challenges within provider experience

#### Providing care in virtual settings

At the outset of the pandemic, many MHP had limited virtual care experience which proved challenging for some. Some MHPs found it difficult to gauge affect and assess risk levels as many CARES users opted for phone calls rather than video communication. This contributed to a heightened sense of worry in some MHPs of “missing something”.

#### Navigating and maintaining boundaries in shared work setting

Given that MHPs and UHN CARES service users belonged to the same institution, there was uncertainty about setting appropriate boundaries to ensure both groups felt comfortable in the therapeutic relationship. This involved discussion of these boundaries as well as acknowledgement of any feelings of role conflict (colleague versus provider) and self-reflection. Some MHPs chose to clarify this upfront in the first interaction with the users, while others did not feel the need to address it at all perceiving the risk of breaching any boundaries as low.

Concerns of HCWs at times proved distressing to MHP as well. Given their shared workplace, there was inherently a greater sense of resonance in the issues raised and at times a sense of the user's experience being "close to" their "own identity".

#### Managing requests, referrals, follow-up care outside of the workplace program

UHN CARES users represented diverse professions (e.g., physicians, research assistants, nurses, spiritual workers, occupational therapists, physiotherapists, respiratory therapists, social workers, dieticians, administrative staff, etc.). There was also a wide variety of concerns expressed, and how those concerns evolved throughout the pandemic. MHPs frequently identified that users required additional support as they transitioned out of the program which was addressed by development of a dedicated referral pathway to community resources.

#### Anticipation of support required

Some MHPs who had never provided care to HCWs expressed initial apprehension, which later resolved as they gained experience within the framework of the program and were able to consult their colleagues for further support. MHPs also reported that CARES users were overall presenting as a population relatively well at baseline, which may have required a different approach to treatment. They also identified that recommended interventions need to be catered to the shorter-term nature of the program.

### Strategies for providing high-quality care

#### Skills and tools for patient-centeredness

Given the short-term nature of the program, MHPs creatively implemented effective ways to equip CARES users with the individualized support that they needed, within the CARES program itself and through community referrals. MHPs noted that this was a patient population that often had a particularly good sense of their needs and were collaborative in developing a treatment plan, including number and pacing of sessions. Attention to transitions, including termination of support was also identified as an important strategy (Box 1 ).

#### Personal coping strategies

With extensive social restrictions, numerous impacts on the healthcare system throughout the pandemic, and the added workload from UHN CARES, MHPs identified that developing ways to cope with their own stressors was important. Some MHPs expressed that providing support to frontline workers was, in fact, something that helped them cope. Others mentioned prioritizing sleep, physical activity, and using strategies to step away from work-related communication.

## Discussion

It is known that mental health supports for frontline workers is a crucial component of institutional emergency preparedness to maintain infrastructure, as navigating through the turmoil of disasters often results in a substantial emotional burden on frontline staff as well as those providing the mental health services [2, 3, 11]. Therefore, in this study we aimed to characterize the experiences of MHPs working within a workplace-based mental health support program throughout the COVID-19 pandemic. The longitudinal nature of this qualitative study enabled us to examine what made for the successful and sustainable delivery of mental health supports to HCWs as the pandemic evolved.

Generally, MHPs described their involvement in UHN CARES to be rewarding as they felt it was their contribution to bolstering the mental resolve of their frontline colleagues. Flexibility, confidentiality, administrative support, access to resources, and adaptation of the program in response to MHP feedback were highlighted as key components contributing to providers’ positive experience. Providers’ dual role as MHP within their routine contexts, as well as pandemic distress responders for colleagues within their organisation was not experienced as burdensome but rather provided an opportunity for meaning-centred coping for providers [21]. This appears to have enabled them to take on the additional program workload while managing the mental health consequences of the pandemic on their regular patients, as well as their own adjustment needs within a global pandemic [22]. This finding is congruent with positive findings in other initiatives led by mental health professionals [7, 11].

All UHN CARES program MHPs were highly skilled clinicians with a range of 2 to 32 years’ work experience, plus many years of prior clinical training. This level of experience helped inform their approach and flexibly adapt to varying HCW support needs and allowed for easy access to more experienced colleagues for consultation and debriefs when needed. Providers without this breadth of training and access to colleagues may be more anxious, have less knowledge, and in turn, feel more burdened by their additional role [11]. Adequate training and sufficient level of prior experience appears to have been protective and may have increased the sense of preparedness within our MHPs [23].

In certain instances, the strengths of UHN CARES doubled as challenges for some MHPs. As both users and providers were affiliated with the same organisation, it often increased relatability, facilitating therapeutic rapport, and giving providers an intimate understanding of the local and institutional stressors and resources. Conversely, this also resulted in moral injury for some providers as they learned about their colleagues’ distressing experiences and institutional challenges. Providers also identified some challenges in working within the limitations of the scope of the program (i.e., short-term interventions solely to members of the UHN team). Establishing clear guidelines and developing a compendium of external resources allowed MHPs to offer distressed users some form of support while still minimizing their feelings of helplessness and moral injury.

Although the program’s flexibility was a major strength, not all providers had the same availability and scope to provide flexible appointments and expressed feelings of guilt as a result. These concerns were balanced by the knowledge that the program was a team effort, not reliant on a sole individual. This highlights the importance of having a large provider pool, offering each one more clinical flexibility. The opportunity for MHP flexibility in the number of new assessments they accepted and when they saw service users enabled providers to self-regulate their workload.

These structural components of UHN CARES may enhance the sustainability of the program while the pandemic continues to impact HCWs, increasing provider satisfaction and potentially mitigating against provider burnout, which is accelerated in conditions of low worker autonomy [24]. Our findings also add to the small literature that exists surrounding the provision of support to HCWs in earlier pandemics, where flexibility was also identified as an important component of successful uptake of support [25].

As would be expected in an institutional-based mental health support program, there is the added challenge of navigating and maintaining professional boundaries as roles between provider and colleague/user can become blurred [11]. Personal strategies to offset this burden included MHP reflexivity and collaborating with the user to acknowledge and plan for potential moments of conflict (e.g., running into each other in an informal environment). Strategies embedded into the program included having administrative staff notify MHPs in advance if a user was from their own department and directing care toward other providers in the pool as well as ensuring confidentiality by storing records separate from UHN’s electronic patient record system, preventing access by others within the institution.

### Limitations

This project was undertaken as a quality improvement initiative with a focus on program evaluation, not as a research project. As such, it was single site and reflective of the practice and culture of HCWs and MHPs at UHN. The emphasis of the analysis was on depth of understanding, rather than broad generalizability. That said, we sampled MHPs with a wide range of experiences to obtain a broader view of the program. A longitudinal interview format enabled us to track how issues evolved and member-check our findings as we developed the thematic analysis, enhancing the reliability of our results. As this program was designed and delivered using a quality improvement framework, feedback was used to rapidly adapt the program and assess the impact of those changes. While the evolving nature of the program itself might pose challenges for determining the impact of programmatic elements, the overall approach was in line with participatory effectiveness factors demonstrated in previous studies on the development of rapid response programs [10, 11].

## Conclusions

As HCW mental health is critical for ensuring high quality patient care and a resilient health system, understanding how workplace support programs can be sustained long-term and the experiences of MHPs working within them is necessary. In addition to insights into the program’s success from the perspective of developing and retaining a sizeable cohort of service providers, we have highlighted strategies and structures that may be applicable in other settings. Developing a body of evidence about how to provide mental health support to HCWs and sustain such programs over time will enable health systems to be better equipped for future crises [11, 26].

## Supplementary Information

**Additional file 1:** **Supplemental Table 1.** Personal Characteristics of Research Team.

## Data Availability

The datasets used and/or analysed during the current study are available from the corresponding author on reasonable request.

## Abbreviations

HCW: Healthcare workers
MHP: Mental healthcare providers
UHN CARES: University Health Network Coping and Resilience for Employees and Staff
UHN: University Health Network
PPE: Personal protective equipment
QIRC: Quality Improvement Review Committee
REB: Research Ethics Board
CR: Critical realism
