# Supplementary material for: Future public health emergencies and disasters: sustainability and insights into support programs for healthcare providers
Source: BMC Psychiatry. 2022 Oct 27;22:664. doi: 10.1186/s12888-022-04309-z (PMC9612620; doi:10.1186/s12888-022-04309-z)
Supplement: Supplementary file 1 — Additional file 1: Supplemental Table 1. Personal Characteristics of Research Team. [file 12888_2022_4309_MOESM1_ESM.docx]

Supplemental Table 1. Personal Characteristics of Research Team

| Initials of Researcher | Credentials | Gender | Occupation | Research Design | Interviewer | Interviewee | Coder | Provider Pool |
| --- | --- | --- | --- | --- | --- | --- | --- | --- |
| MD | MPH | F | Medical Student | ✓ | ✓ |  | ✓ |  |
| LR | PhD | F | Psychologist |  |  | ✓ |  | ✓ |
| LM | MD, PhD | F | Psychiatrist |  |  | ✓ |  | ✓ |
| MM | PhD | F | Psychologist |  |  | ✓ |  | ✓ |
| AL | DClinPsy | F | Psychologist |  |  | ✓ |  | ✓ |
| DS | MD | F | Psychiatrist |  |  |  |  | ✓ |
| SA | MD | F | Psychiatrist |  |  | ✓ |  | ✓ |
| SB | MD, PhD | F | Psychiatrist | ✓ | ✓ | (✓)* | ✓ | ✓ |
| KS | MD, DPhil | F | Psychiatrist | ✓ | ✓ |  | ✓ | ✓ |
| CSQ | MD | M | Psychiatrist | ✓ |  | ✓ |  | ✓ |

* SB served as initial interviewee as part of the piloting phase of interview schedule
